# Investigating the additive effects of Alzheimer’s disease biomarker staging and subjective cognitive decline in the prediction of clinical progression

**DOI:** 10.1002/alz.091377

**Published:** 2025-01-03

**Authors:** Melina Stark, Michael Wagner, Elizabeth Kuhn, Katharina Bürger, Emrah Düzel, Julian Hellmann‐Regen, Michael T. Heneka, Christoph Laske, Robert Perneczky, Oliver Peters, Josef Priller, Matthias Schmid, Anja Schneider, Annika Spottke, Stefan Teipel, Jens Wiltfang, Frank Jessen, Luca Kleineidam

**Affiliations:** ^1^ Department of Neurodegenerative Diseases and Geriatric Psychiatry, University of Bonn Medical Center, Bonn Germany; ^2^ German Center for Neurodegenerative Diseases (DZNE), Bonn Germany; ^3^ Institute for Stroke and Dementia Research (ISD), University Hospital, LMU, Munich Germany; ^4^ German Center for Neurodegenerative Diseases (DZNE), Munich Germany; ^5^ German Center for Neurodegenerative Diseases (DZNE), Magdeburg Germany; ^6^ Institute of Cognitive Neurology and Dementia Research (IKND), Otto‐von‐Guericke University, Magdeburg Germany; ^7^ German Center for Neurodegenerative Diseases (DZNE), Berlin Germany; ^8^ Charité – Universitätsmedizin Berlin, corporate member of Freie Universität Berlin and Humboldt‐Universität zu Berlin – Institute of Psychiatry and Psychotherapy, Berlin, Berlin Germany; ^9^ German Center for Mental Health (DZPG), Berlin Germany; ^10^ Luxembourg Centre for Systems Biomedicine (LCSB), University of Luxembourg, Luxembourg Luxembourg; ^11^ German Center for Neurodegenerative Diseases (DZNE), Tuebingen Germany; ^12^ Section for Dementia Research, Hertie Institute for Clinical Brain Research and Department of Psychiatry and Psychotherapy, University of Tuebingen, Tuebingen Germany; ^13^ LMU University Hospital, Munich Germany; ^14^ Munich Cluster for Systems Neurology (SyNergy), Munich Germany; ^15^ Department of Psychiatry and Psychotherapy, University Hospital, LMU Munich, Munich Germany; ^16^ Charité – Universitätsmedizin Berlin, corporate member of Freie Universität Berlin and Humboldt‐Universität zu Berlin – Institute of Psychiatry and Psychotherapy, Berlin Germany; ^17^ School of Medicine, Technical University of Munich; Department of Psychiatry and Psychotherapy, Munich Germany; ^18^ University of Edinburgh and UK DRI, Edinburgh United Kingdom; ^19^ Department of Psychiatry and Psychotherapy, Charité, Berlin Germany; ^20^ Institute of Medical Biometry, Informatics and Epidemiology, University Hospital Bonn, Bonn Germany; ^21^ Department of Neurology, University of Bonn, Bonn Germany; ^22^ German Center for Neurodegenerative Diseases (DZNE), Rostock Germany; ^23^ Department of Psychosomatic Medicine, University of Rostock, Rostock Germany; ^24^ German Center for Neurodegenerative Diseases (DZNE), Goettingen Germany; ^25^ Department of Psychiatry and Psychotherapy, University Medical Center, University of Goettingen, Goettingen Germany; ^26^ Neurosciences and Signaling Group, Institute of Biomedicine (iBiMED), Department of Medical Sciences, University of Aveiro, Aveiro Portugal; ^27^ Excellence Cluster on Cellular Stress Responses in Aging‐Associated Diseases (CECAD), University of Cologne, Cologne Germany; ^28^ Department of Psychiatry, University of Cologne, Medical Faculty, Cologne Germany

## Abstract

**Background:**

The identification of cognitively unimpaired individuals at risk of short‐term cognitive decline is a critical task for Alzheimer´s disease (AD) research. Cognitively normal individuals with amyloid and/or tau pathology have a high risk for short‐term cognitive decline. However, not all of these individuals show clinical progression. Individuals in clinical stage 2 of AD, characterized e.g. by subjective cognitive decline (SCD), are thought to be temporally closer to clinical progression to mild cognitive impairment (MCI), but this hypothesis has not been rigorously investigated yet.

**Method:**

We included 195 memory clinic SCD patients and 83 cognitively normal participants without SCD (CN) with baseline CSF and longitudinal cognitive data from the observational DELCODE study. Participants were categorized into three AD biomarker stages (A‐T‐, A+T‐, A+T+) based on their baseline CSF Aβ42/40 and p‐tau_181_ status. The groups were compared in their longitudinal preclinical Alzheimer´s cognitive composite (PACC5) trajectories and average time until progression to MCI. Group differences in the time until progression to MCI, over eight years of follow‐up, were estimated with restricted mean survival time models. All analyses were adjusted for demographic covariates.

**Result:**

Compared to the A‐T‐ group (62 CN, 123 SCD), A+T‐ (16 CN, 49 SCD) and A+T+ (5 CN, 23 SCD) participants showed significantly accelerated PACC5 decline and a faster progression to MCI (A+T‐: 2.7 [‐5.7‐11.2] and A+T+: 22.4 [8.1‐36.8] months earlier than A‐T‐;). SCD patients had a significantly faster PACC5 decline and progression to MCI (15.5 [9.8‐21.1] months) than CN participants. The effects of SCD and the biomarker stages on both outcome measures remained significant when the predictors were entered in the same models. In exploratory analyses, SCD patients tended to show a faster progression to MCI than CN participants within the same biomarker group (A+T‐: 19.0 [6.3‐31.7] and A+T+: 14.0 [‐20.8‐48.8] months earlier than CN in same biomarker group).

**Conclusion:**

SCD provides incremental information for the identification of individuals at high risk of imminent cognitive decline beyond a biomarker‐based classification of AD pathology. In cognitively unimpaired individuals, the clinical stage should be taken into account additionally to AD biomarkers for an improved prediction of the time until clinical progression.

